# New Appliances and Things Medical

**Published:** 1902-12-13

**Authors:** 


### NEW APPLIANCES AND THINGS MEDICAL.

[We shall be glad to receive at our Office, 28 & 29 Southampton Street, Strand, London, W.C., from the manufacturers, specimens of all new preparations and appliances which may be brought out from time to time.]

### VALENTINE'S MEAT JUICE.

(Mann S. Valentine, Richmond, Va., U.S.A.)

Since Valentine's meat juice was placed upon the market many years ago, innumerable imitations have been introduced to the public in the hope that they might supplant this well-known and highly-appreciated preparation in the affection of the public and of the medical world. No stronger evidence of the intrinsic merit of this meat juice can be adduced than that in so many cases of real emergency from threatening inanition, it is on Valentine that dependence is placed. The value of a proteid diet in the treatment of disease is now almost universally recognised in all cases of wasting disease. No other form of food is capable of repairing tissue waste. Of all proteid foods, the animal proteids contained in meat juice perform this service with the greatest certainty and efficiency. And Valentine's meat juice is itself one of the most reliable of all known preparations of this kind.

### AGURIN: A NEW DIURETIC.

(The Bayer Co., Limited, Elberfeld, and 19 St. Dunstan's Hill, London, E.C.)

Agurin is a white powder, highly soluble in water, and very hydroscopic. It has a bitter saline taste, and chemically must be regarded as a double salt of sodium—theobromine and acetate of soda. Its therapeutic action is very powerfully diuretic. This new salt is one of the latest products of rational therapeutic chemistry. The object in preparing this double salt was to find a substitute for diuretin which should possess the same remarkable power of inducing diuresis, and at the same time should be free from the objection which attaches to this latter substance in that it contains a large proportion of salicylic acid, which in some conditions is contra-indicated for continued administration. Acetate of soda has been substituted for the salicylic radicle contained in diuretin. Our personal experiences of the action of diuretin in cases of dropsy from cardiac inefficiency and hepatic cirrhosis encourages us to believe that any double salt, such as Agurin, which possesses even more powerful diuretic effects than diuretin and is free from the objections which must, unfortunately, be urged against the continued use of this latter drug, must have a great future before it.

### BYROLIN.

(Dr. Graf and Co., 25 Cheapside, London, E.C.)

The Byrolin preparations consist of "Byrolin," an antiseptic toilet cream, and three varieties of soap, namely, 1. cakes of Byrolin soap; 2. Byrolin paste soap in tubes; 3. Byrolin liquid soap in bottles. We have not so far had an opportunity of examining the Byrolin soaps; however, as regards the ointment, we can confirm from personal experience the high opinion which has already been expressed by medical men on the Continent with regard to its application in dermatological practice, and for the lubrication of catheters and other instruments. As a simple ointment for general use we can highly commend it.

### ÆSCULAP WATER.

(The Æsculap Bitter Water Company, London, E.C., and Budapest).

Among the natural sulphated mineral waters "Æsculap' has for many years held a prominent position, and most deservedly so, for medical men, who are not swayed and influenced by passing fancies in the same way that the public are, continue to prescribe these waters in virtue of their constant and uniform chemical constitution, and by reason of the exemplary care which is not only exercised in the preservation of the springs themselves from contamination, but also in the bottling, transit, and storage of the water itself. Chemical analysis of the water proves that it is peculiarly free from organic contamination, and that its mineralisation consists for the most part of the sulphate of sodium, magnesium, and calcium, with smaller percentages of the sulphates of potassium and ammonia, and of traces of various carbonates and chlorides. As a natural aperient water, Æsculap has the advantage of being less unpleasant to drink than many other waters which are taken with the same therapeutic object: its complex mineralisation is happily adjusted to the physiological requirements of many persons suffering from chronic constipation and uric acid poisoning. As an aperient the dose is about 5 ounces, as a corrective for acidity a smaller dosage after meals will be found to meet the requirements of the case.

